# High-Resolution Ultrasonography of Renal Oncocytoma Presenting with Symptomatic Hematuria and Urinary Bladder Clot Retention—A Rare Occurrence

**DOI:** 10.15586/jkcvhl.v9i1.200

**Published:** 2022-01-01

**Authors:** Reddy Ravikanth

**Affiliations:** Department of Radiology, St. John’s Hospital, Bengaluru, India

**Keywords:** high-resolution ultrasonography, renal oncocytoma, symptomatic hematuria, urinary bladder clot retention

## Abstract

Renal oncocytomas are asymptomatic, benign tumors often encountered incidentally on various imaging modalities. Renal oncocytomas comprise 5–7% of primary renal neoplasms and are derived from cells of the distal renal tubule. We present a case report of renal oncocytoma in a 22-year-old male having right-sided flank pain and symptomatic gross hematuria with a giant urinary bladder clot retention. The tumor was excised, and the patient underwent laparoscopic partial nephrectomy. Typical features of renal oncocytoma were observed upon histopathological examination of the resected specimen. The patient was catheterized, and bladder irrigation with clot retraction was performed.

## Introduction

Renal oncocytoma was first described by Zippel in 1942 as a benign tumor of the renal parenchyma (1). They are usually solitary and constitute 3–10% of all renal tumors (2). Renal oncocytoma has a strong male predilection with mean age of presentation being sixth to seventh decades of life (3). Mostly renal oncocytomas are solitary and unilateral but may be bilateral in 4–5% of cases, and multifocality may be seen in 13% of the cases (4). Renal oncocytomas are benign, asymptomatic, small lesions which cannot be reliably distinguished from renal cell carcinomas (RCC) preoperatively (5). Classically, renal oncocytoma is a solid mass lesion with a central fibrous scar arising from the renal parenchyma. Most cases of renal oncocytoma are discovered only incidentally during routine imaging evaluation of nonurological problems and are often asymptomatic at presentation, but flank pain, hematuria, and a palpable mass may be encountered in a minority of documented cases. The variable nature of presentation and overlap of radiological findings between renal oncocytoma and RCC may often complicate their clinical differentiation. Due to the benign clinical course of renal oncocytoma, usually long-term outcomes have been excellent. The disease-specific survival rate of patients with renal oncocytoma is 100% (6).

## Case Report

**Figure 1:**
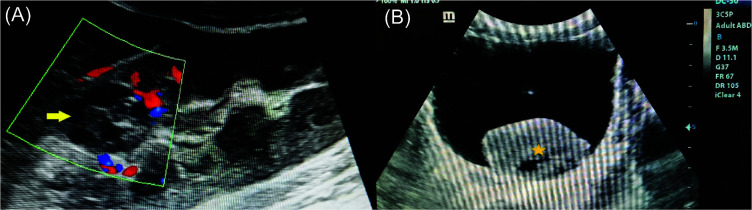
Color Doppler ultrasonography image demonstrating no significant vascularity within the well-circumscribed heteroechoic cortical based lesion (arrow) in the upper pole of right kidney. (A) High-resolution transverse ultrasonography image demonstrating a giant retained clot (star) within the lumen of the urinary bladder secondary to symptomatic hematuria (B).

**Figure 2:**
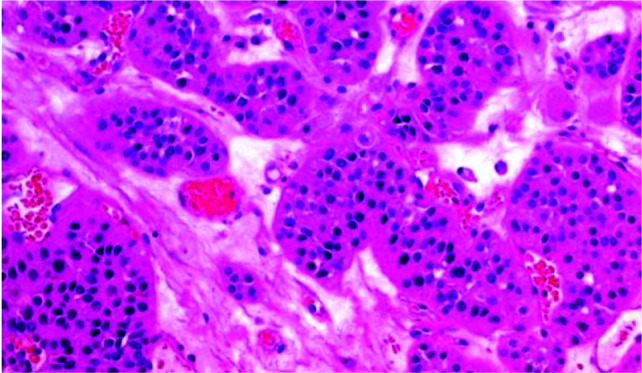
Histopathology section of the excised specimen demonstrating round and polygonal cells packeted within a loose stromal background, eosinophilic and finely granular cytoplasm, round nuclei with inconspicuous nucleoli, and absent or rare mitotic figures consistent with a diagnosis of renal oncocytoma (H and E, × 400).

A 22-year-old male presented to the Department of Emergency Medicine with complaints of sudden onset right flank pain accompanied by gross hematuria. Pain was moderate in intensity, nonradiating with no history of trauma or fever. Vitals including pulse, blood pressure, and respiratory rate were within normal limits. Urine microscopy demonstrated multiple red blood cells suggesting hematuria. The patient was referred to the Department of Radiology for ultrasonography of the abdomen which revealed a well-circumscribed, heteroechoic, cortical based lesion measuring 2.0 × 1.8 cm located in the upper pole of right kidney. Color Doppler demonstrated no significant internal vascularity within the lesion (Figure 1A). Furthermore, a well-defined heteroechoic mass was noted at the dependent portion of the urinary bladder, suggestive of a giant retained clot (Figure 1B). Chest radiograph, chest computed tomography (CT), and bone scans were all negative for metastasis. Based on the radiological findings, a diagnosis of renal oncocytoma with symptomatic hematuria leading to giant clot retention in the urinary bladder was made. The patient was referred to the Department of Urology for further management where she underwent laparoscopic partial nephrectomy of the right kidney and tumor resection. The resection margins were free of tumor, and there was no evidence of perinephric invasion or lymphadenopathy. Histopathological examination of the resected specimen revealed round and polygonal cells within a loose stromal background, eosinophilic granular cytoplasm, round nuclei with inconspicuous nucleoli, and absent or rare mitotic figures consistent with a diagnosis of renal oncocytoma (Figure 2). The patient was further catheterized, and urinary bladder irrigation with clot retraction was performed. The patient recovered well with no complications and was discharged home in good condition on the seventh day post operation. At 3 months follow-up, the patient was free of symptoms and had no signs of recurrence.

The patient has given written informed consent to publish his case and clinical images.

## Discussion

Renal oncocytomas are the most common benign renal tumors constituting 3–7% of tumors affecting the kidney (7). Definitive preoperative diagnosis of renal oncocytoma is challenging as laboratory and clinical findings usually reveal no specific characteristics of the entity. While contrast-enhanced computed tomography (CECT) scan may demonstrate a well-circumscribed, solid lesion with homogeneous contrast enhancement lesion and presence of a centrally located scar, arteriography may demonstrate a spokewheel vascular pattern of renal oncocytoma. On cross-sectional imaging, solid enhancing renal lesions need to be differentiated from RCC. However, a central stellate scar with segmental enhancement and a spoke-wheel pattern of feeding arteries are common imaging characteristics of a renal oncocytoma.

Renal oncocytoma should be included in the differential diagnosis of solid renal mass lesions discovered incidentally or tumors located within a solitary kidney. Although benign in nature, occasionally renal oncocytomas can reach the size of giant renal mass with associated pressure symptoms on adjacent viscera. Renal oncocytomas are generally well-circumscribed lesions and are rarely invasive or associated with distant metastases. Rarely, renal oncocytoma may integrate with adjacent perinephric fat and may invade renal vein, thus making differentiation from RCC difficult. In our case, partial nephrectomy was indicated for relief of symptomatic hematuria due to this hypervascular renal tumor.

Central stellate scar and a spoke-wheel pattern of feeding arteries in renal oncocytomas are unreliable diagnostic signs for preoperative differential diagnosis and are of poor predictive value (8). Hence, enhancing solid renal lesions should be treated as RCC with curative intent depending on the clinical circumstances. Coexisting RCC has been reported in 10–32% of patients with renal oncocytomas. Hence, timely surveillance and close monitoring of renal oncocytoma are necessary to look for evidence of rapid growth (9).

Radical or partial nephrectomy is performed in majority of patients with renal oncocytoma, based on the clinical complaints such as gross hematuria. It has been advocated that in patients with renal oncocytoma, with tumors <4 cm in size located in the upper or lower poles of the kidney may be treated with a partial nephrectomy, while in patients with tumors >4 cm in size or located in the mid-pole may require a radical nephrectomy to be performed (10). However, considering the benign behavior of renal oncocytomas, partial nephrectomy is a more appropriate treatment option compared with radical nephrectomy.

Renal oncocytomas have a benign clinical course with excellent long-term outcomes (11). Surgical management of solid renal tumors is still unclear. Although radical surgery has been employed in the past as primary treatment for renal tumors, more precise diagnosis of benign solid renal tumors at preoperative and perioperative stages should allow for more frequent use of conservative treatment strategies, such as partial nephrectomy. Conservative management such as organ-sparing surgery or partial nephrectomy should be reserved for bilateral renal tumors or when the tumor occurs in a solitary kidney (12).

Symptomatic cases usually present with hematuria, loin pain, or palpable mass. There had been reports of bilateral and multifocal oncocytomas (13), oncocytomas in pregnancy (14), as well as associations with other renal neoplasms such as RCC (9) and angiomyolipoma (15). However, there were no earlier reported cases of renal oncocytoma with gross hematuria in patients with no syndromic association or collision tumors. Our case report deserves a special mention, as it demonstrates massive hemorrhage from a renal lesion to the extent of forming a giant clot within the urinary bladder and highlights the importance of vigilant follow-up of these benign renal lesions, as very rarely they may be associated with serious complications.

## Conclusion

Renal oncocytomas are asymptomatic parenchymal lesions with benign clinical course and excellent long-term outcomes. Occasionally, symptomatic hematuria leading to urinary bladder clot retention may be encountered, which may complicate the clinical course of renal oncocytoma. However, the combination of clinical, radiological, and histopathological features may assist in lesion characterization of renal oncocytoma and differentiation from RCC. Partial nephrectomy is still considered the most appropriate treatment for majority of patients with renal oncocytomas.
